# Supplementary material for: In depth analysis of Cyprus-specific mutations of SARS-CoV-2 strains using computational approaches
Source: BMC Genom Data. 2021 Nov 13;22:48. doi: 10.1186/s12863-021-01007-9 (PMC8590444; doi:10.1186/s12863-021-01007-9)
Supplement: Supplementary file 2 — Additional file 2. [file 12863_2021_1007_MOESM2_ESM.docx]

**
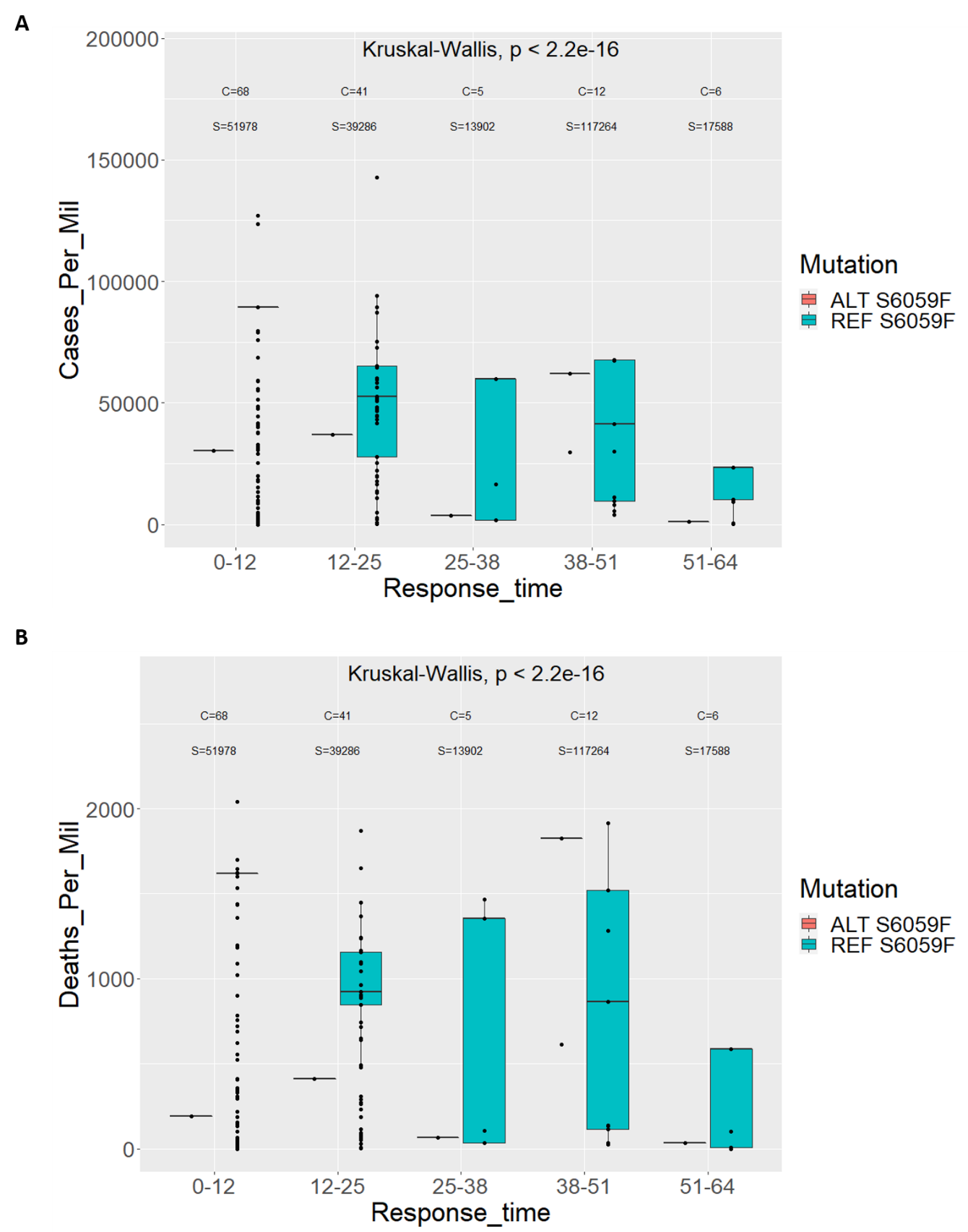
**

**Figure S1 A.** Deaths per million for countries with the S6059F mutation and the reference mutation including response time separation. *C* denotes the number of unique countries in the group and *S* is the number of strains in the group. **B.** Cases per million for countries with the P13L mutation and the reference mutation including response time separation. *C* and *S* are as denoted for panel **A**.


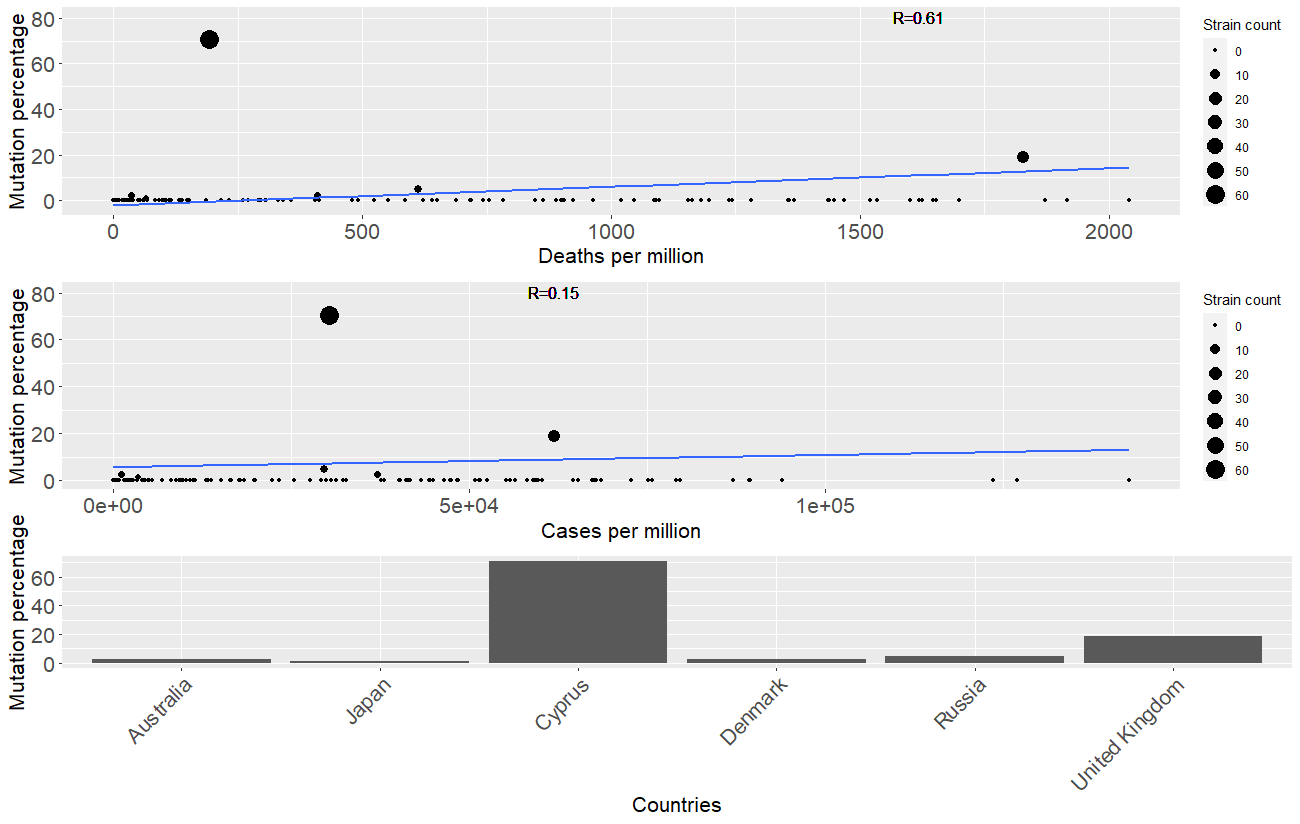


**A**

**B**

**C**

**Figure S2. Analyses plots for mutation S6059F** **A**. Regression model line showing the simplified fit for mutations percentage across countries and the deaths per million for each country. Pearson’s correlation is shown by the *R* value. **B**. Similar regression fit for mutations percentage across countries this time showing cases per million for each country. Pearson’s correlation is shown by the *R* value. **C**. Detailed histogram of the percentage occurrence of the mutation across different countries. These values make up the “percent” predictor variable utilized in our model (for details see *Oulas* *et al,* PLoS One. 2021 Jan 26;16(1):e0238665. doi: 10.1371/journal.pone.0238665). Countries are sorted with increasing deaths per million.
